# Reduction of renal interstitial fibrosis by targeting Tie2 in vascular endothelial cells

**DOI:** 10.1038/s41390-023-02893-8

**Published:** 2023-11-27

**Authors:** Lu Jiang, Xiaohan Hu, Yajun Feng, Zhen Wang, Hanyun Tang, Qiang Lin, Yunyan Shen, Yun Zhu, Qinying Xu, Xiaozhong Li

**Affiliations:** 1 Department of Nephrology and Immunology, Children’s Hospital of Soochow University, Suzhou, 215003 China; 2 Institute of Pediatrics, Children’s Hospital of Soochow University, Suzhou, 215003 China; 3 Department of Pediatrics, Jiangyin People’s Hospital, Jiangyin, 214400 China; 4 Department of Pediatrics, Zibo Maternal and Child Health Care Hospital, Zibo, 255000 China

## Abstract

**Background:**

Tie2, a functional angiopoietin receptor, is expressed in vascular endothelial cells and plays an important role in angiogenesis and vascular stability. This study aimed to evaluate the effects of an agonistic Tie2 signal on renal interstitial fibrosis (RIF) and elucidate the underlying mechanisms.

**Methods:**

We established an in vivo mouse model of folic acid-induced nephropathy (FAN) and an in vitro model of lipopolysaccharide-stimulated endothelial cell injury, then an agonistic Tie2 monoclonal antibody (Tie2 mAb) was used to intervent these processes. The degree of tubulointerstitial lesions and related molecular mechanisms were determined by histological assessment, immunohistochemistry, western blotting, and qPCR.

**Results:**

Tie2 mAb attenuated RIF and reduced the level of fibroblast-specific protein 1 (FSP1). Further, it suppressed vascular cell adhesion molecule-1 (VCAM-1) and increased CD31 density in FAN. In the in vitro model, Tie2 mAb was found to decrease the expression of VCAM-1, Bax, and α-smooth muscle actin (α-SMA).

**Conclusions:**

The present findings indicate that the agonistic Tie2 mAb exerted vascular protective effects and ameliorated RIF via inhibition of vascular inflammation, apoptosis, and fibrosis. Therefore, Tie2 may be a potential target for the treatment of this disease.

**Impact:**

- This is the first report to confirm that an agonistic Tie2 monoclonal antibody can reduce renal interstitial fibrosis in folic acid-induced nephropathy in mice.
- This mechanism possibly involves vascular protective effects brought about by inhibition of vascular inflammation, apoptosis and fibrosis.
- Our data show that Tie2 signal may be a novel, endothelium-specific target for the treatment of tubulointerstitial fibrosis.

## Introduction

Renal tubulointerstitial fibrosis is an important factor in the progression of chronic kidney disease (CKD).^1,2^ An increasing number of animal and clinical studies are reporting that tubulointerstitial fibrosis is associated with peritubular capillary loss.^3–6^ In one such study, Yuan et al.^7^ demonstrated that peritubular capillary loss may result in tubulointerstitial fibrosis in folic acid-induced nephropathy (FAN) mice. Consequently, protecting damaged renal microvasculature is a crucial approach to ameliorating renal interstitial fibrosis, and angiogenesis may play an important role in the process.^8,9^

Tie2 is a transmembrane receptor tyrosine kinase found almost exclusively on endothelial cells. Angiopoietin-1 (Ang-1) is a endogenous ligand for Tie2 that can activate Tie2. The binding of Tie2 by Ang-1 triggers pathways that promote angiogenesis and regulate vascular stability.^10–14^ Previous reports have shown that Ang-1/Tie2 signaling improves endothelial survival, downregulates inflammatory pathways,^15,16^ and rescues apoptosis.^17,18^ Recent studies have found that dysregulation of Ang-1/Tie2 signaling is a significant feature in patients with CKD.^19^ Interestingly, previous research has reported contradictory results about the effect of Ang-1 in renal fibrosis. For example, while a soluble, stable, and potent Ang1 variant (COMP-Ang1) was found to protect peritubular capillaries, downregulate inflammation and delay fibrotic changes in a unilateral ureteral obstruction (UUO) mouse model.^20^ Contrary to the findings observed in UUO models, administration of a soluble form of Ang-1 was found to enhance fibrosis and inflammation in a FAN mouse model.^21^ In previous studies, Yuan et al. have demonstrated that the agonistic Tie2 monoclonal antibody (Tie2 mAb) stimulates Tie2 activation,^22^ which maintains the integrity of recently formed interstitial vessels.^23^ Further, Tie-2-expressing capillaries were found to undergo proliferation in the fibrotic interstitium between atrophic tubules in a mouse model of FAN.^24^ In the present study, we have tried to expand this line of research by exploring the potential renoprotective effects of Tie2 mAb.

In this study, the in vivo FAN mouse model and the in vitro lipopolysaccharide (LPS)-stimulated human umbilical vein endothelial cell (HUVEC) injury model were used to verify that exogenous treatment with Tie2 mAb can improve tubulointerstitial lesions and decrease tubulointerstitial fibrosis by reducing peritubular capillary inflammation and upregulating peritubular capillary density. Our in vivo and in vitro findings indicate that Tie2 mAb may have therapeutic applications in the treatment of CKD.

## Methods

### Animal experiments: the FAN model and Tie2 mAb treatment

**Fig. 1:**
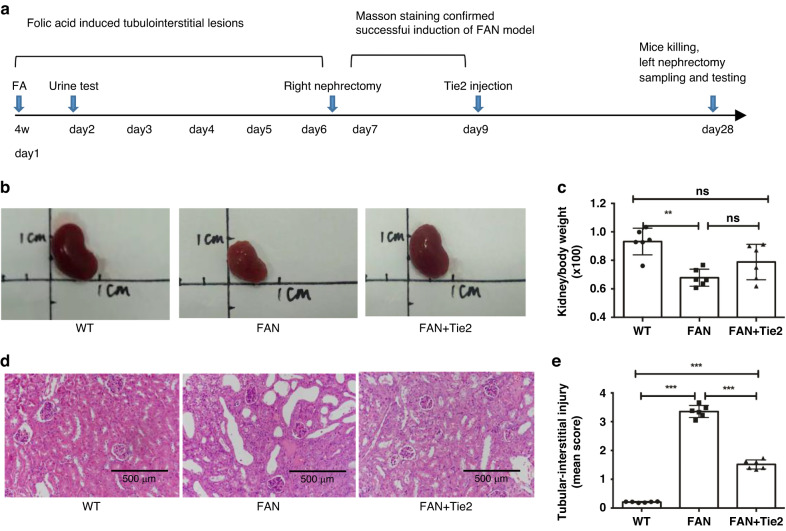
Attenuation of renal atrophy and lesions in FAN mice with Tie2 mAb treatment. **a** FA or vehicle only was administered on day 1. Urine samples were tested for determining UACR on day 2. Right nephrectomy was performed for routine histological analysis by Masson staining on day 7. FAN+Tie2 group mice were intraperitoneally administered 1 mg Tie2 mAb on day 9. All the mice were sacrificed on day 28. **b** Renal appearance was evaluated and (**c**) kidney/body weight were measured to assess renal atrophy. **d** HE staining was performed to evaluate the histopathological changes in murine renal tissues (magnification, ×200). **e** Renal lesions were evaluated by calculating the mean tubular-interstitial injury score. All data are presented as mean ± SD (*n* = 6). *p < 0.05, **p < 0.01, ***p < 0.001. FA folic acid, FAN folic acid nephropathy, Tie2 mAb agonistic Tie2 monoclonal antibody, UACR urinary albumin-to-creatinine ratio (mg/mg).

Male CD1 mice (*n* = 18; age, 4 weeks old) weighing 14–18 g were purchased from Suzhou Sinosure Biotechnology Co. Ltd. (Suzhou, China). The mice were randomly divided into three groups (*n* = 6 per group): WT, FAN, and FAN+Tie2. On day 1, mice of the FAN and FAN +Tie2 groups were intraperitoneally administered FA (F7876, 240 mg/kg; Sigma, Saint Louis) in vehicle (0.2 ml of 0.3 mol/L NaHCO_3_). The FA dose used has been previously shown to reliably induce severe nephrotoxicity.^24^ Mice of the WT group were only intraperitoneally administered vehicle. Urine samples were tested for determining the urinary albumin-to-creatinine ratio (UACR) on day 2. Right nephrectomy was performed on each of the mice to obtain tissue for routine histological analysis by Masson staining on day 7. The results revealed FA-induced renal injury, as evidenced by a high uACR and renal pathological changes. Mice in the FAN+Tie2 group were treated by intraperitoneal injection of 1 mg of Tie2 mAb (AF313; R&D, Minneapolis) on day 9. The mice were sacrificed by cervical dislocation, and left nephrectomy was performed for renal tissue studies on day 28 (Fig. 1a).

### In vitro experiments: LPS-induced HUVEC injury model and Tie2 mAb treatment

HUVECs were obtained from Procell Life Science and Technology Co. Ltd. (Wuhan, China) and were kept at 37 °C in a 5% CO_2_-containing humidified incubator. Dulbecco’s modified Eagle’s medium (DMEM; Sigma, Saint Louis) supplemented with 10% fetal bovine serum (FBS; Gibco, New York) was used as the culture medium. HUVECs were incubated in a 5% CO_2_ incubator at 37 °C, and the medium was replaced every 2–3 days. The cells were then exposed to LPS (10 µg/ml; L4005, Sigma, Saint Louis) for construction of the in vitro model. Following this, HUVECs with LPS-induced injury were treated with Tie2 mAb (5 µg/ml; AF313, R&D, Minneapolis).

### Hematoxylin–eosin and Masson staining

Masson staining was performed using the standard procedure as described previously.^25^ At 7 days after FA injection, the right kidneys of the mice were freshly dissected, fixed, processed, and embedded in paraffin. The kidney sections were cut to a thickness of 3 mm, and the sections were stained with Masson’s trichrome. At 28 days after FA injection, the left kidneys were freshly dissected, fixed, processed, and embedded in paraffin. Then, 3-mm sections were cut and stained with hematoxylin-eosin (HE) and Masson’s trichrome. To observe the pathological changes in renal tissues, the sections were subjected to HE staining and scored on a scale from 0 to 4 (0: no changes, 1: changes affecting <25% of the section, 2: changes affecting 25–50% of the section, 3: changes affecting 50–75% of the section; and 4: changes affecting 75–100% of the section).^26^ The tissue sections were visualized and photographed under a light microscope (Olympus Corporation, Tokyo, Japan). Tubulointerstitial fibrotic areas were evaluated by Masson’s trichrome. Using the Olympus Cell Soft Imaging System, the area of tubulointerstitial fibrosis and the total area of each visual field were measured and their ratio was calculated. Then, the mean of all the values was calculated.

### Immunohistochemistry staining

At 28 days after FA injection, the left kidneys of the mice were preserved in 4% paraformaldehyde. The tissues were then embedded in paraffin, and transverse paraffin sections (thickness, 5 mm) were mounted on silane-coated slides. The sections were deparaffinized, rehydrated, and then incubated with the FSP1 antibody (1:100; ab41532, Abcam, Cambridge, UK) or CD31 antibody (1:100; ab124432, Abcam, Cambridge, UK). Immunolabelled sections were visualized with 0.05% DAB plus 0.3% H2O2 in phosphate-buffered saline.

### Western blot analysis

Protein was extracted from renal tissues and HUVECs using radioimmunoprecipitation assay lysis buffer (Beyotime Institute of Biotechnology, Shanghai, China) containing 1% phenylmethylsulfonyl fluoride (Beyotime Institute of Biotechnology, Shanghai, China). The Enhanced BCA Protein Assay kit (Beyotime Institute of Biotechnology, Shanghai, China) was used to determine protein concentration. Proteins were separated by sodium dodecyl sulfate-polyacrylamide gel electrophoresis and transferred onto polyvinylidene fluoride membranes. The membranes were probed with primary antibodies against VCAM-1 (1:2000; ab134047, Abcam, Cambridge, UK) or α-SMA (1:2500; ab32575, Abcam, Cambridge, UK). Western blot analysis for specific protein expression was performed according to established procedures as described previously.^27^

### RNA isolation and quantitative PCR

RNA was isolated from HUVECs by using the standard protocol of the TRIzol manufacturer. cDNA was reverse-transcribed by using Cell Counting Kit-8 (Japan Tonin Co. Ltd., Japan). The following primers against VCAM-1 and Bax were used: VCAM-1, 5′-CCTGCTGGGATATTAGCTCCA-3′ (forward) and 5′-CAGCGGTAGGTGTCGAAGC-3′ (reverse); Bax, 5′-GTGCACCAAGGTGCCGGAAC-3′ (forward) and 5′-TCAGCCCATCTTCTTCCAGA-3′ (reverse). The thermocycling conditions were as follows: denaturation for 5 min at 95 °C followed by 40 cycles of 10 s at 94 °C, 20 s at 60 °C, and 30 s at 72 °C. The relative mRNA levels were calculated using the 2-ΔΔCq method.^28^

### Statistical analysis

GraphPad Prism Version 6 (GraphPad Prism Software Inc., San Diego, California) was used for data analysis and figure preparation. Results were expressed as mean ± SEM. One-way analysis of variance (ANOVA) was used to compare the means of the three groups. *p* < 0.05 was considered to indicate significance.

## Results

### Attenuation of renal atrophy and lesions in FAN model mice treated with Tie2 mAb

On day 2, urine samples from FAN model mice were collected for determining UACR, which is an established marker for renal dysfunction.^29^ UACR was higher than normal in the model mice. On day 7, the right kidneys were resected for routine histological analysis by Masson staining. Tubular damage was detected 1 d after FA administration. Over the next few weeks, most tubules regenerated but exhibited patchy atrophy, and interstitial fibrosis was also observed.^24^ Consistent with these findings, the present results revealed FA-induced renal injury, as evidenced by high UACR and renal pathological changes (Fig. S1a, b).

We divided the mice into three groups: WT mice, FAN model mice, and FAN model mice treated with the agonistic Tie2 mAb. Figure 1 shows the changes induced in FAN mice treated with Tie2 mAb. The mice were intraperitoneally administered 1 mg of Tie2 mAb on day 9 (Fig. 1a). Visual examination by autopsy of the FAN mice revealed renal shrinkage with uneven surfaces, and a lower kidney/body weight ratio than the WT group (*p* < 0.01). In the FAN mice treated with Tie2 mAb, the degree of kidney shrinkage was reduced (Fig. 1b) and the kidney/body weight ratio was substantially improved compared with the FAN mice that did not receive this treatment (Fig. 1c, Table 1). These data suggest that treatment with Tie2 mAb ameliorated renal atrophy in FAN mice. We further evaluated histopathological changes in kidney tissue by HE staining. A considerable number of tubular casts and severe cell infiltration, tubular atrophy, and interstitial fibrosis were detected in the FAN mice; these effects were suppressed by Tie2 mAb administration (Fig. 1d). In addition, the tubulointerstitial injury score was decreased after Tie2 mAb treatment in the FAN mice (*p* < 0.001) (Fig. 1e). These findings suggest that treatment with Tie2 mAb mitigated FA-induced tubulointerstitial lesions in mice.

**Table 1:** Kidney weight and body weights.

Group	Kidney weight (g)	Body weight (g)	Kidney/body weight ratio (×100)
WT	0.33 ± 0.02	35.61 ± 2.00	0.93 ± 0.09
FAN	0.23 ± 0.02	34.08 ± 1.86	0.68 ± 0.06
FAN+Tie2	0.26 ± 0.05	33.35 ± 5.19	0.79 ± 0.12
*F*	21.160	0.299	7.538
*p*	0.003	0.608	0.019

### Suppression of renal tubulointerstitial fibrosis in FAN mice treated with Tie2 mAb

**Fig. 2:**
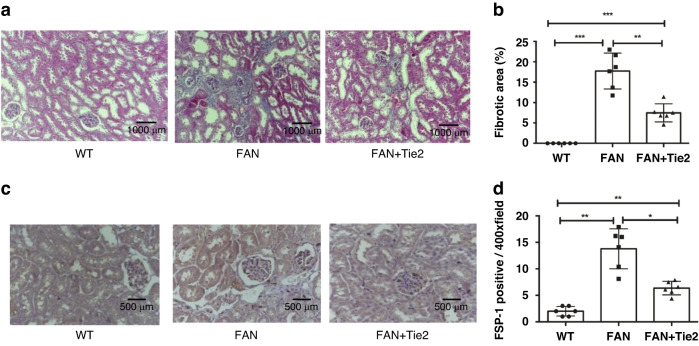
Suppression of renal tubulointerstitial fibrosis in FAN mice by Tie2 mAb treatment. **a** Masson staining was performed to evaluate tubulointerstitial fibrosis in murine renal tissues (magnification, ×200). **b** Semiquantitative score of tubulointerstitial fibrosis in Masson’s trichrome-stained sections. Ten randomly selected high-power fields were quantified and the average value was calculated for each mouse. **c** Expression of FSP1 in murine renal tissues was assessed by immunohistochemical staining (magnification, ×400). **d** The number of infiltrating FSP1-positive cells in kidneys was counted. All data are expressed as mean ± SD (*n* = 6). *p < 0.05, **p < 0.01, ***p < 0.001. FSP1 fibroblast-specific protein 1.

Masson staining was performed to observe collagen deposition and renal interstitial fibrosis. Significantly increased interstitial collagen deposition was observed on day 28 in the FAN mice, but this effect was suppressed by Tie2 mAb administration in the antibody-treated FAN mice (Fig. 2a). Treatment with Tie2 mAb resulted in a marked reduction in the FA-induced tubulointerstitial fibrosis area (*p* < 0.01) (Fig. 2b). In addition, the expression of FSP1 in renal tissues was evaluated by immunohistochemical staining (Fig. 2c). FSP1 has been shown to be correlated with serum creatinine levels, creatinine clearance, and fibrosis area as confirmed by renal biopsy, and is a predictor of end-stage renal disease.^30^ A significant increase in FSP1 staining in renal tissues was observed in the FAN mice on day 28, whereas Tie2 mAb treatment effectively inhibited FA-induced expression of FSP1 (*p* < 0.05) (Fig. 2d).

### Attenuation of inflammation and improved density of peritubular capillaries in FAN mice treated with Tie2 mAb

**Fig. 3:**
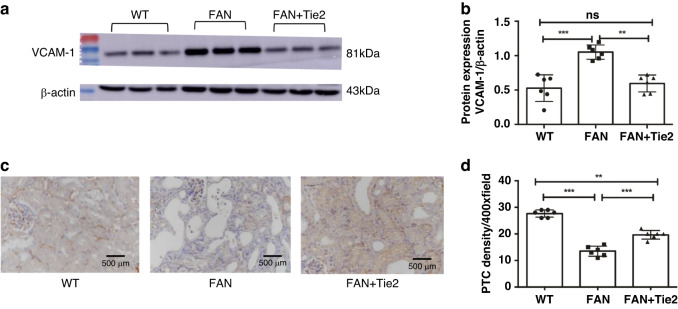
Attenuation of inflammation and improved density of peritubular capillaries in FAN mice by Tie2 mAb treatment. **a** Protein expression level of VCAM-1 in murine renal tissues was assessed by western blotting. **b** Densitometry results are presented as the relative ratio of VCAM-1 to β-actin. **c** The density of PTC was evaluated by immunohistochemical staining of CD31 (magnification, ×400). **d** Density of PTC in the kidneys. All data are expressed as mean ± SD (*n* = 6). *p < 0.05, **p < 0.01, ***p < 0.001. VCAM-1 vascular cell adhesion molecule-1, PTC peritubular capillaries, CD31 cluster of differentiation 31.

The expression of VCAM-1, an important mediator of vascular inflammation, was assessed by western blotting (Fig. 3a). Elevated expression of VCAM-1 was observed in the FAN mice, but this effect was diminished on treatment with Tie2 mAb (*p* < 0.01) (Fig. 3b). This indicates that Tie2 mAb had an anti-inflammatory effect. Furthermore, immunohistochemical staining of CD31, a typical marker of endothelial cells (Fig. 3c), showed that peritubular capillaries could be easily observed by CD31 immunostaining. In the FAN mice, a decrease in the number of CD31-positive capillaries was observed in renal tissues, but this effect was mitigated on administration of Tie2 mAb (*p* < 0.001) (Fig. 3d). These results suggest that treatment with Tie2 mAb prevented decrease in the density of peritubular capillaries in FAN mice.

### In vitro protective effect of Tie2 mAb on vascular endothelial cells

**Fig. 4:**
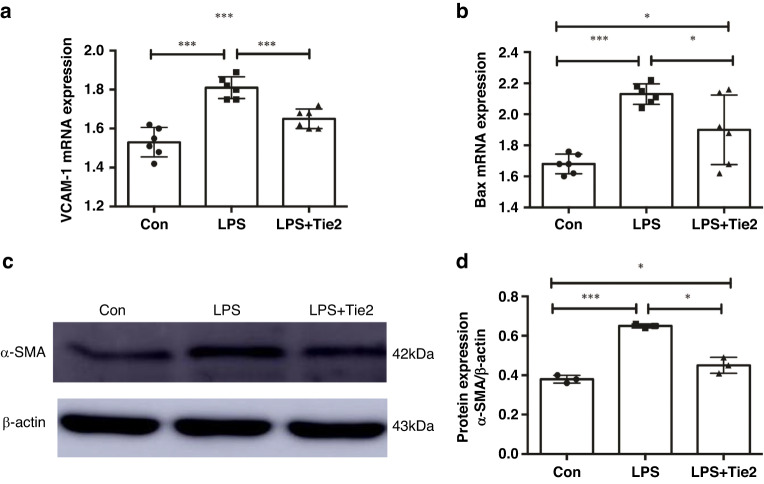
In vitro protective effect of Tie2 mAb against damage to vascular endothelial cells. **a** mRNA levels of VCAM-1 and (**b**) Bax were detected by quantitative PCR. **c** Protein expression level of α-SMA in HUVECs was assessed by western blotting. **d** Densitometric analyses were presented as the relative ratio of α-SMA to β-actin. All data are expressed as mean ± SD (*n* = 6). *p < 0.05, **p < 0.01, ***p < 0.001. VCAM-1 vascular cell adhesion molecule-1, Bax Bcl-2-associated X protein, α-SMA α-smooth muscle actin, HUVECs human umbilical vein endothelial cells.

Based on the ameliorative effect of Tie2 mAb on peritubular capillary lesions and renal interstitial fibrosis in the mouse model of FAN, we used HUVECs with LPS-induced injury as an in vitro model to visualize and quantify the effect of Tie2 mAb on vascular endothelial cells. The mRNA expression levels of the inflammatory factor VCAM-1 (Fig. 4a) and the apoptosis gene Bax (Fig. 4b) in the LPS group were significantly higher than those in WT group (*p* < 0.05), while those in Tie2 mAb treatment group were significantly lower than those in the LPS group (*p* < 0.05). This suggests that Tie2 mAb had anti-inflammatory and anti-apoptotic effects on injured HUVECs. Thus, Tie2 mAb promoted endothelial cell regeneration.

Next, the in vitro effect of Tie2 mAb on the expression of the pro-fibrotic factor α-SMA was examined in HUVECs. As shown in Fig. 4c, d, the protein expression level of α-SMA was significantly increased on stimulation with LPS in HUVECs. However, this increase in α-SMA levels was significantly suppressed by treatment with Tie2 mAb (*p* < 0.05). These results demonstrate that Tie2 mAb treatment resulted in a weakened fibrotic response in endothelial cells.

## Discussion

The folic acid-induced nephropathy (FAN) mouse model is a classical model for acute kidney injury (AKI) and sequential tubulointerstitial fibrosis. FA induces dose-dependent nephrotoxicity in mice, accompanied with acute tubular epithelial apoptosis.^24^ Endothelial cell injury in FA-induced nephropathy involves damage to tubular epithelial cells as a result of the production of proinflammatory cytokines, such as NF-kB, TNF-α, and IL-6.^31–33^ That it, endothelial cell injury is not directly caused by FA. Eventually, this leads to peritubular capillary lesions and tubulointerstitial fibrosis in FAN model mice. Similarly, we used LPS as an inflammatory agent to establish an in vivo model of endothelial cell injury in HUVECs. LPS is one of the most common proinflammatory stimuli that promotes the production of various inflammatory cytokines.^34^ The FAN model and LPS-induced injury model are analogous to each other. To demonstrate the successful induction of the FAN model, we performed right nephrectomy to obtain tissue for Masson staining and evaluate the tubulointerstitial lesions in murine renal tissues on day 7. This was also performed in the WT group mice. It should be noted that the removal of one kidney did not cause tubulointerstitial lesions at this time.

Our data demonstrate for the first time that administration of Tie2 mAb ameliorates renal tubulointerstitial fibrosis, dampens renal inflammation, and promotes the growth of peritubular capillaries after FA-induced renal injury in a mouse model. The potential protective effect of this antibody on endothelial cells was confirmed through in vitro experiments on HUVECs with LPS-induced injury, and the findings were in line with previous studies that have reported the protective effect of Ang-1/Tie2 activation on endothelial cells.^15–18^ Recently, Carota et al. ^35^ suggested that activation of Tie2 by inhibition of vascular endothelial protein tyrosine phosphatase reduces the expression of pro-inflammatory and pro-fibrotic gene targets in diabetic nephropathy. Rübig et al. ^36^ also provided evidence that the in vivo activation of Tie2 by PEGylated VT, a drug-like Tie2 receptor agonist, can counteract microvascular endothelial barrier dysfunction, improve renal recovery, and reduce mortality in ischemic acute kidney injury. Here, we used a reliable model of CKD to study the impact of Tie2 mAb on kidney fibrosis. In accordance with these previous findings, we found that Tie2 mAb treatment prevented FA-induced renal vascular inflammation, improved the density of peritubular capillaries, and significantly attenuated interstitial fibrosis.

In the present study, we observed that the accumulation of FSP1 positive cells and the interstitial fibrotic area were significantly decreased by Tie2 mAb treatment. The findings indicate that Tie2 mAb had an anti-fibrotic effect. Consistent with these findings, the in vitro protein expression level of α-SMA, a putative marker of myofibroblasts,^37^ was increased in HUVECs with LPS-induced injury, while it was markedly repressed by treatment with Tie2 mAb. In addition, the anti-inflammatory effect of Tie2 mAb was demonstrated by downregulation of VCAM-1 expression. In accordance with these findings, there is increasing evidence that Tie2 activation is associated with anti-inflammatory effects in endothelial cells,^38,39^ and that Tie2 mAb inhibit endothelial VCAM-1 expression. In addition, in the present study, we examined LPS-induced apoptosis in endothelial cells as evidenced by elevated levels of the proapoptotic gene Bax.^40^ Interestingly, Tie2 mAb administration enhanced endothelial cell survival in response to apoptotic injuries.^41^ Hence, Tie2 mAb inhibited inflammation, rescued apoptosis, and suppressed fibrosis. The preservation of peritubular capillaries by Tie2 mAb treatment is a potential future research direction to further explore the mechanism of Tie2 mAb in the treatment of CKD or improving renal fibrosis.

There is increasing evidence to show that peritubular capillaries play an important role in CKD and are a key regulator of CKD progression.^4^ Peritubular capillary rarefaction is found not only in diabetic nephropathy^42^ and hypertensive nephropathy,^43^ but also in IgA nephropathy,^44^ congenital nephrotic syndrome,^45^ lupus nephritis,^46^ and polycystic kidney disease.^47^ Thus, protecting peritubular capillaries is a crucial approach to alleviating renal fibrosis.^48–51^ In the present study, we observed that Tie2 mAb administration in FAN mice is associated with an increase in the density of peritubular capillaries. This effect might be due to a Tie2 activation-induced elevation in endothelial cell number that directly enhances endothelial cell proliferation^52^ or prevents endothelial cells apoptosis.^53^ We were unable to explore the mechanisms in detail in the present study, so more research is needed to explore the signaling pathway involved in renal tubulointerstitial fibrosis and how it is affected by the administration of Tie2 mAb.

## Conclusions

In summary, we confirmed that the agonistic Tie2 mAb can ameliorate renal interstitial fibrosis by reducing vascular inflammation and improving the density of peritubular capillaries in FAN model mice. With regard to the underlying mechanisms, the in vitro results indicated that Tie2 mAb ameliorates capillary lesions by inhibiting inflammation, rescuing apoptosis, and suppressing fibrosis. The results of this study provide new insights into reversing renal fibrosis through the vascular protective effects of Tie2 mAb.

## Availability of data and materials

All data generated or analysed during this study are included in this published article.

### Supplementary information

Figure S1

Figure S1 legend
