# Supplementary figures and images for: Reduction of renal interstitial fibrosis by targeting Tie2 in vascular endothelial cells
Source: Pediatr Res. 2023 Nov 27;95(4):959–65. doi: 10.1038/s41390-023-02893-8 (PMC10920200; doi:10.1038/s41390-023-02893-8)

a

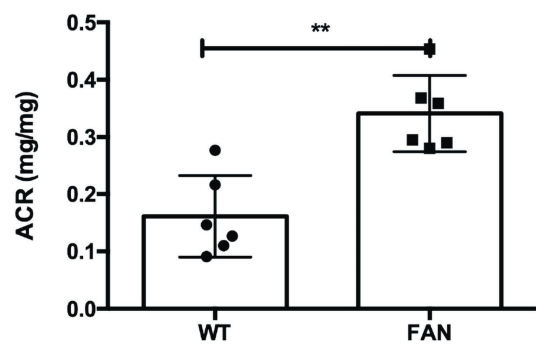

b

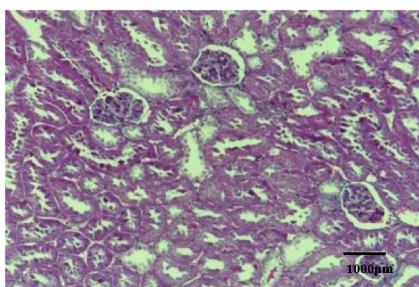

WT

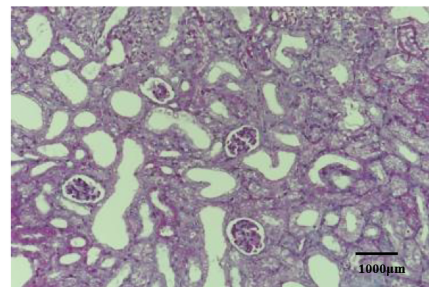

FAN

Supplement: Supplementary file 1 — Figure S1 [file 41390_2023_2893_MOESM1_ESM.pdf]
