# Supplementary material for: Reduction of renal interstitial fibrosis by targeting Tie2 in vascular endothelial cells
Source: Pediatr Res. 2023 Nov 27;95(4):959–65. doi: 10.1038/s41390-023-02893-8 (PMC10920200; doi:10.1038/s41390-023-02893-8)
Supplement: Supplementary file 2 — Figure S1 legend [file 41390_2023_2893_MOESM2_ESM.pdf]

Fig. S1 Evidence for successful induction of the FAN mouse model

(a) Urine samples were tested for determining UACR on day 2. FAN mice showed higher UACR than WT mice. (b) Right nephrectomy was performed to obtain tissue for histological analysis by Masson staining (magnification, × 200) on day 7. Tubulointerstitial lesions were revealed in FAN murine renal tissues. All data are expressed as mean ± SD (n = 6). $*p < 0.05$, $**p < 0.01$. FAN, folic acid-induced nephropathy; UACR, urinary albumin-to-creatinine ratio (mg/mg).
